# The endangered California Condor (*Gymnogyps californianus*) population is exposed to local haemosporidian parasites

**DOI:** 10.1038/s41598-020-74894-0

**Published:** 2020-10-21

**Authors:** M. Andreína Pacheco, Chris N. Parish, Timothy J. Hauck, Roberto F. Aguilar, Ananias A. Escalante

**Affiliations:** 1 Biology Department/Institute of Genomics and Evolutionary Medicine (iGEM), Temple University (SERC - 645), 1925 N. 12th St., Philadelphia, PA 19122-1801 USA; 2 The Peregrine Fund, 5668 West Flying Hawk Lane, Boise, ID 83709 USA; 3 Tucson Wildlife Center, 13275 East Speedway, Tucson, AZ 85748-7125 USA

**Keywords:** Ecology, Evolution, Genetics, Microbiology, Molecular biology

## Abstract

The endangered California Condor (*Gymnogyps californianus*) is the largest New World Vulture in North America. Despite recovery program success in saving the species from extinction, condors remain compromised by lead poisoning and limited genetic diversity. The latter makes this species especially vulnerable to infectious diseases. Thus, taking advantage of the program of blood lead testing in Arizona, condor blood samples from 2008 to 2018 were screened for haemosporidian parasites using a nested polymerase chain reaction (PCR) protocol that targets the parasite mitochondrial cytochrome b gene. *Plasmodium homopolare* (Family Plasmodiidae, Order Haemosporida, Phylum Apicomplexa), was detected in condors captured in 2014 and 2017. This is the first report of a haemosporidian species infecting California Condors, and the first evidence of *P. homopolare* circulating in the Condor population from Arizona. Although no evidence of pathogenicity of *P. homopolare* in Condors was found, this study showed that the California Condors from Arizona are exposed to haemosporidian parasites that likely are spilling over from other local bird species. Thus, active surveillance should be an essential part of conservation efforts to mitigate the impact of infectious diseases, an increasingly recognized cause of global wildlife extinctions worldwide, particularly in avian populations considered vulnerable or endangered.

## Introduction

The endangered California Condor (*Gymnogyps californianus*) is the largest avian scavenger in North America; this New World Vulture has an average wingspan of 2.8 m and a bodyweight of 8.5 kg^1^. The species was distributed in western North America and Florida before the late Pleistocene megafaunal extinctions about 10,000 years ago^2^. However, by the nineteenth century, it was restricted mainly to the West Coast, from British Columbia to Baja California. The populations declined until it practically became extinct in the wild around 1987, prompting the removal of the remaining 22 individuals to breeding facilities. Then, release efforts initiated in 1992, succeeded by increasing the population to over 250 birds by 2005, and to more than 500 total by 2017^3–5^.

Condors were first reintroduced to California; however, in the context of the federal recovery plan, the US Fish and Wildlife Service and The Peregrine Fund established a captive breeding facility in Boise, Idaho, followed by a release program in northern Arizona. In 1996, releases began there to create a self-sustaining disjunct population^6^. Continuous releases brought the number of free-flying birds to about 99 by spring 2020, including eighteen from wild pairs within northern Arizona and southern Utah (C. Parish personal communication, April 12, 2020). At present, the California Condor has stable managed populations in the south and central California, northern Arizona, southern Utah, and Baja California (Mexico)^6–8^. Although this is a successful recovery effort, there has been a substantial loss of genetic diversity over a relatively short period (> 80% reduction in unique haplotypes over the past two centuries)^9^. As a result of this population bottleneck, inbreeding has increased, and a decreased fitness has been observed in the captive-bred population^9–11^.

Nowadays, daily monitoring of radio-tagged condors by conventional very high frequency (VHF) and satellite-based Global Positioning System (GPS) telemetry is used to follow its distribution range from the Grand Canyon National Park to the Zion region of southern Utah. The opportunistic recovery of condor carcasses allows assessing the various mortality agents. The most frequent cause of diagnosed death has been lead poisoning from the ingestion of lead ammunition residues (bullet fragments, intact bullets, and lead shot) in the remains of gun-killed animals^4,6,12,13^. In 2000, at least two individuals died from ingesting shotgun pellets from an unknown source, and thirteen others showed elevated blood lead levels in Arizona^14^. This event, followed by a general expansion of condor movement and foraging in the region^15^, prompted the development of a regular program of blood lead testing, evaluation, and treatment.

With known limited genetic variability and increased morbidity and mortality from lead poisoning, additional stresses to condors caused by infectious diseases could further hamper recovery efforts. In particular, environmental stress has been positively correlated with *Plasmodium* prevalence, the agents of avian malaria. Thus, endangered avian populations should be monitored for Haemosporida as part of their health assessments. Regardless of the ongoing research efforts on avian haemosporidian parasites, information about the species that infect New World Vultures is still limited^16–20^. Species of *Haemoproteus*, *Plasmodium* and/or *Leucocytozoon* have been only reported in Turkey Vulture, Black Vulture, and King Vulture^18,19,21–25^; however, some of those studies lack molecular data and there is no data from the California Condor.

Taking advantage of the program of blood lead testing in Arizona, this study reports a molecular surveillance screening for haemosporidian parasites on specimens collected during the winter from 2008 to 2018. This is the first report of a haemosporidian species infecting California Condors, specifically the first evidence of a lineage of *Plasmodium homopolare* infecting the Condor population from Arizona.

## Results

### Molecular diagnostic of haemosporidian parasites

Out of 208 condor blood samples screened for haemosporidian parasites, one condor from 2014 (582) and two from 2017 (561 and 605) were positive by nested PCR (3/208, 1.4%). None of the positive condors were detected by the primary PCR indicating that the parasitemia could be very low (< 0.01), consistent with a subpatent infection. All three sequences obtained here were 100% identical to *P. homopolare* (e.g., 100% similarity with *P. homopolare* JN792148^26^) using BLAST^27^.

All three positive condors were males in good health based on appearance and vigor despite one, condor 605, having high lead levels upon recapture. Indeed, no evidence of pathogenicity of *P. homopolare* in condors was found. A positive condor (582) collected in 2014 hatched in May 2010 and was released in February 2012. Interestingly, this individual was recaptured in 2016, 2017, and 2018 and it was negative by nested PCR (Supporting information Table S1). Condor 561 hatched in April 2010 and was released in March 2012, and condor 605 hatched in April 2011 and was released in December 2012. Both condors were negative by nested PCR in 2014 and 2015, suggesting that the infection occurred later in the study area (around 2016–2017). Unfortunately, condors 561 and 605 were not recaptured in the 2018-trapping season; however, telemetry data indicated that they were still alive at the time of concluding the screening. Indeed, it is common that not every bird is captured every year as the population becomes more independent of the Release Site.

### Phylogenetic and population analyses

**Figure 1:**
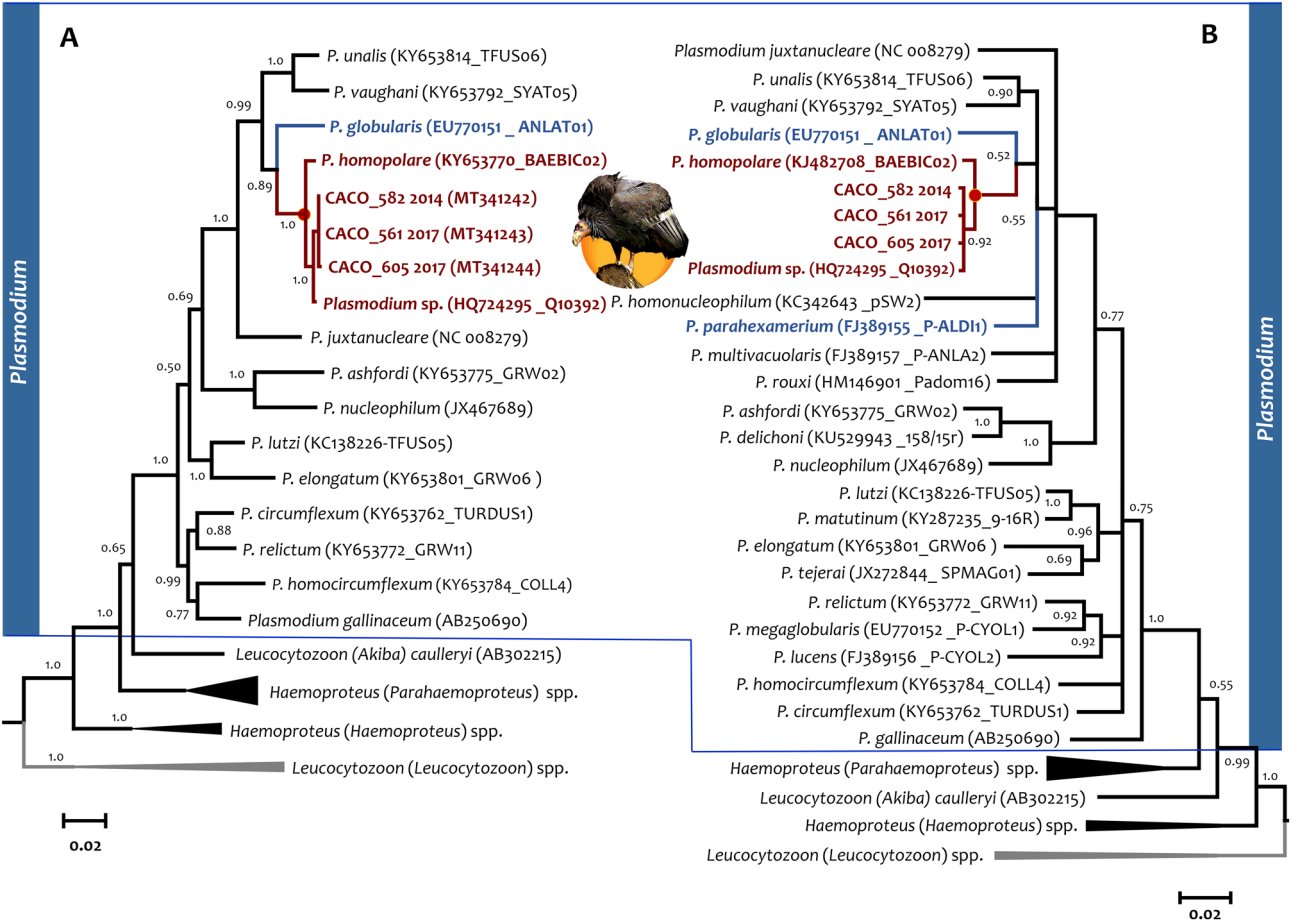
Bayesian phylogenetic hypotheses of *Plasmodium* parasites infecting the California Condors (*Gymnogyps californianus*) from Arizona, USA. Phylogenetic trees were computed based on parasites (**A**) partial sequences of the *cytb* gene (35 sequences and 1,012 out of 1,134 bp) and (**B**) the commonly used *cytb* gene fragment (45 sequences and 464 out of 1,134 bp). The values above branches are posterior probabilities. *Leucocytozoon* genus (outgroup) is indicated in grey. The lineage identifiers, as deposited in the MalAvi database, and their Genbank accession numbers are provided in parenthesis for the sequences used in the analyses. *Plasmodium homopolare* haplotypes are indicated in red and *P. globularis* and *P. parahexamerium*, the closely related parasite to *P. homopolare*, are indicated in blue. Condor silhouette was designed by Ariana Cristina Pacheco Negrin.

Two gene trees were estimated, one (Fig. 1A) with a larger fragment of *cytb* sequences (1,012 out of 1,134 bp, N = 35), and the other (Fig. 1B) with the commonly *cytb* fragment (464 bp of 1,134 bp, N = 45) used to identify haemosporidian parasites^28^. Phylogenetic analyses place all California Condor parasite sequences within a large clade containing all *Plasmodium* parasites. The tree obtained using the larger fragment (Fig. 1A) had better support in most clades, highlighting the importance of using longer *cytb* fragments than those traditionally used when using phylogenetic methods. Both phylogenetic hypotheses indicated that the parasite circulating in the condor population belongs to one of the *P. homopolare* haplotypes (H2, see below) that has been reported previously^26,29^. In both trees, *Plasmodium globularis* seems to be one of the closest taxa to *P. homopolare*. Indeed, the evolutionary divergence between *Plasmodium* species (N = 12) available until now in the databases indicated that *P. parahexamerium* (0.037–0.039) and *P. globularis* (0.039–0.041) are closely related to *P. homopolare* (Table 1).

**Table 1:** Estimates of evolutionary divergence between *Plasmodium* parasites sequences.

		Evolutionary divergence between sequences (standard error estimate)													
		1	2	3	4	5	6	7	8	9	10	11	12	13	14
1	*P. homopolare* (KJ482708_H1)^a^		**0.004**	**0.004**	**0.004**	**0.004**	**0.004**	**0.008**	**0.009**	0.009	0.010	0.010	0.010	0.011	0.011
2	CC582_2014 (MT341242_H2)^b^	**0.006**		**0.000**	**0.000**	**0.000**	**0.000**	**0.009**	**0.009**	0.008	0.009	0.010	0.010	0.011	0.011
3	CC561_2017 (MT341243_H2)^b^	**0.006**	**0.000**		**0.000**	**0.000**	**0.000**	**0.009**	**0.009**	0.008	0.009	0.010	0.010	0.011	0.011
4	CC605_2017 (MT341244_H2)^b^	**0.006**	**0.000**	**0.000**		**0.000**	**0.000**	**0.009**	**0.009**	0.008	0.009	0.010	0.010	0.011	0.011
5	*P. homopolare* (JN792148_H2)^c^	**0.006**	**0.000**	**0.000**	**0.000**		**0.000**	**0.009**	**0.009**	0.008	0.009	0.010	0.010	0.011	0.011
6	*P. homopolare* (HQ724295_H2)^d^	**0.006**	**0.000**	**0.000**	**0.000**	**0.000**		**0.009**	**0.009**	0.008	0.009	0.010	0.010	0.011	0.011
7	*P. parahexamerium* (FJ389155)	**0.037**	**0.039**	**0.039**	**0.039**	**0.039**	**0.039**		**0.009**	0.009	0.009	0.010	0.010	0.011	0.011
8	*P. globularis* (EU770151)	**0.039**	**0.041**	**0.041**	**0.041**	**0.041**	**0.041**	**0.041**		0.009	0.010	0.010	0.010	0.010	0.012
9	*P. unalis* (KY653814)	0.043	0.037	0.037	0.037	0.037	0.037	0.041	0.050		0.008	0.009	0.010	0.011	0.011
10	*P. vaughani* (KY653792)	0.047	0.045	0.045	0.045	0.045	0.045	0.041	0.047	0.032		0.010	0.011	0.011	0.011
11	*P. rouxi* (HM146901)	0.047	0.045	0.045	0.045	0.045	0.045	0.058	0.056	0.043	0.056		0.010	0.011	0.011
12	*P. multivacuolaris* (FJ389157)	0.056	0.058	0.058	0.058	0.058	0.058	0.052	0.047	0.058	0.063	0.054		0.011	0.012
13	*P. juxtanucleare* (NC_008279)	0.058	0.060	0.060	0.060	0.060	0.060	0.058	0.054	0.060	0.069	0.063	0.058		0.012
14	*P. homonucleophilum* (KC342643)	0.067	0.065	0.065	0.065	0.065	0.065	0.063	0.075	0.060	0.065	0.073	0.088	0.082	

The number of base differences per site between sequences are shown and the standard error estimates are shown above the diagonal. The analysis involved 13 nucleotide sequences belonged to Fig. 1B. In bold are indicated the closest species to *P. homopolare* and its haplotypes (H1 or H2).

^a^Sequence obtained from *Melospiza melodia* (passerine bird).

^b^Sequence obtained from *Gymnogyps californianus* (non-passerine bird).

^c^Sequence obtained from *Catharus ustulatus* (passerine bird).

^d^Sequence obtained from *Colinus virginianus ridgwayi* (non-passerine bird).

**Figure 2:**
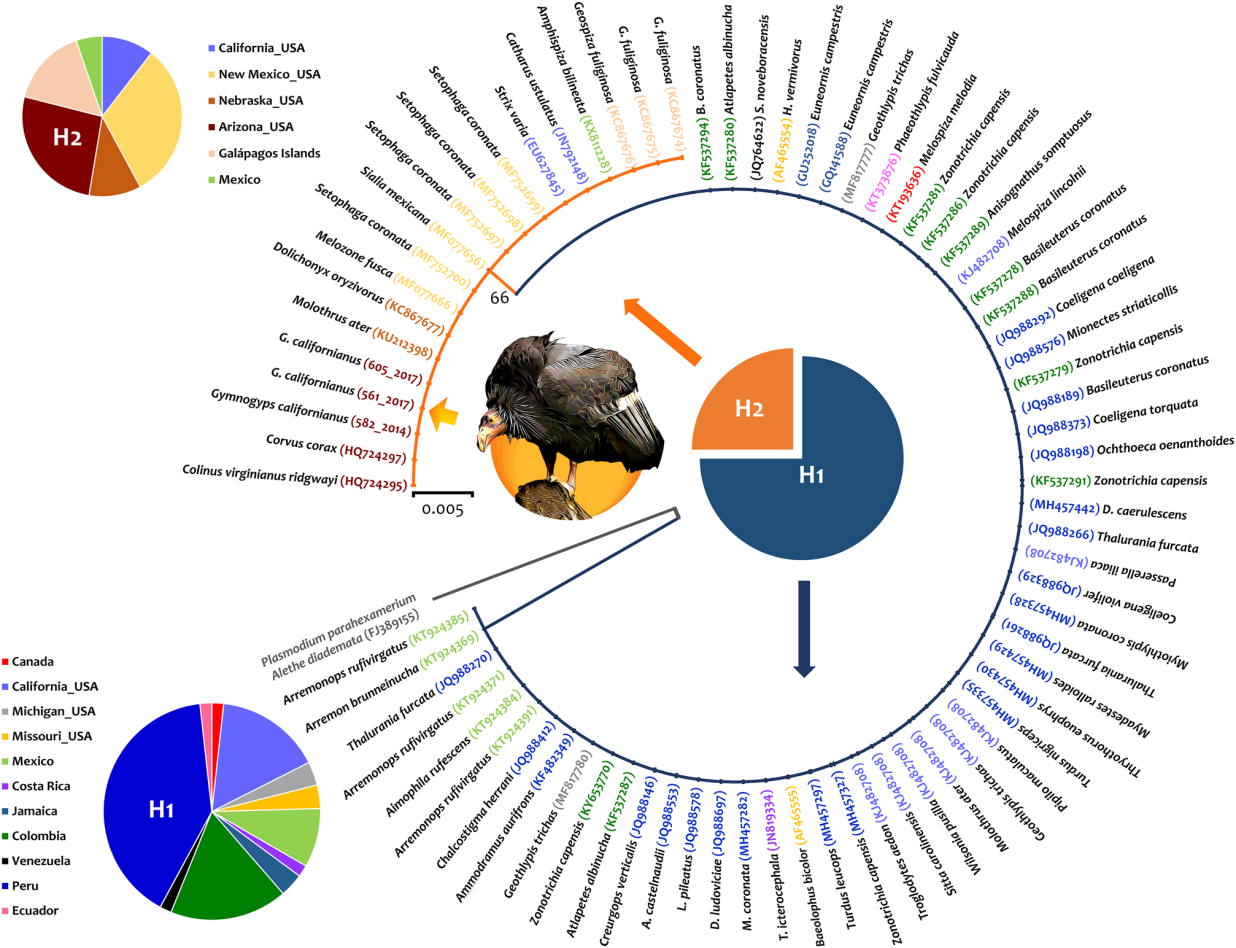
A Maximum Likelihood phylogenetic hypotheses of *P. homopolare* haplotypes infecting California Condors (*Gymnogyps californianus*) from Arizona, USA. Phylogenetic trees were computed based on the sequences obtained from Condors blood samples and the *P. homopolare* haplotypes available on GenBank and MalAvi databases. The parasite host names (indicated by the color of the origin site), and their sequence Genbank accession numbers are shown. The two pie charts indicate the frequency of haplotypes H1 (in blue) and H2 (in orange) per locality. The origins of the haplotypes are identified by color in the pie charts. Condor silhouette was designed by Ariana Cristina Pacheco Negrin.

The relationship between the sequences obtained from condor samples and the *P. homopolare* haplotypes available in databases is shown in Fig. 2. Two haplotypes of *P. homopolare* were found in this analysis (H1 and H2), one of the haplotypes (H1, n = 57, 75%) is mostly distributed in The Americas, and the other (H2, n = 19, 25%) has been only reported in California, New Mexico, Nebraska, Arizona, Mexico, and the Galapagos Islands. The haplotype H2 found in the California Condor is commonly circulating in Arizona (5/19, 26.3%) and New Mexico (6/19, 31.6%). Haplotype H1 has been only found infecting different passerine species and some species of the Apodiformes (Trochilidae family; Fig. 2, Supporting information Tables S1 and S2); most of the passerines are species of the Parulidae, Passerellidae and Thraupidae families (Supporting information Table S3). However, in addition to some passerine species, haplotype H2 has been found infecting, with sign of disease, the endangered species *Colinus virginianus ridgwayi* (Family Odontophoridae, Order Galliformes) in Arizona^30^ and *Strix varia* in California (Strigiformes^31^) (Supporting information Table S2). Genetic distance between the two haplotypes was 0.002 (standard error estimate = 0.002), so only two synonymous substitutions between haplotypes H1 and H2 were found in this *cytb* fragment (464 bp) as has been reported before with fewer data^26^.

## Discussion

To our knowledge, this is the first report of a *Plasmodium* species infecting California Condors. *Plasmodium* (*Novyella*) *homopolare* is a species recently described using microscopy and molecular data (based on 100% or near-100% matches of ≤ 2-nucleotide differences between sequences;^26,29^). Only haplotype H2 of *P. homopolare* was found infecting the Condors (Fig. 2). This haplotype was reported as *Plasmodium* spp. in Common Raven (*Corvus corax*) and Northern bobwhites (*C. virginianus ridgwayi*) from Phoenix, Arizona^30^, and in Northern Barred Owl (*S. varia*) from California^31^, so this is the fourth report of this parasite infecting a non-passerine bird. Overall, the parasite reported here (Figs. 1, 2) has been found infecting a wide range of Passeriformes across The Americas (Supporting information Table S3) with no reports outside the new world. Interestingly, in California (USA), *P. homopolare* has been described as a host generalist parasite with usually light parasitemias, infecting more than 20% of the sampled passerine bird community^26,29^. This pattern may be the result, at least in part, of sampling bias since most of the studies on avian haemosporidian parasites have been done on Passeriformes given the ease of being caught in mist nets.

Based on the few reported studies conducted in The Americas, *Plasmodium* species found in vulture species^18,19,24,32^ exhibit marked differences in prevalence and disease, from the absence of any clinical signs to high mortality^32,33^. These signs depend on host susceptibility and acquired immunity, host fitness status at the time of infection, environmental stress, and parasite species and host genetics, among others^32,33^. Until now, there are some parasites like *Plasmodium relictum, Plasmodium elongatum, Plasmodium circumflexum, Plasmodium matutinum* and *Plasmodium vaughani* that appear commonly associated with disease, and they are worth particular attention in avian health^32,33^. There is no report of these parasites infecting New World Vultures. However, *P. relictum* has been linked to the death of five individuals of Bearded Vultures (*Gypaetus barbatus,* Accipitridae family) in Aragon, Spain (^34^, U. Höfle personal communication, March 31, 2020). The Bearded Vulture is considered as near threatened by IUCN Red List of Threatened Species. According to the study carried out on *G. barbatus* (“*Fundación para la conservación del Quebrantahuesos*,” 2020), the new incidence of *P. relictum* in the area is hypothesized to be linked to climate change (Gonzalez-Serrano et al.^34^, U. Höfle personal communication, March 31, 2020). Although the Bearded Vultures is not a true vulture species, it provides an example of how exposure to a vector-borne parasite, in that case, a virulent species, can affect host populations when it is infecting an immunologically naïve species under some environmental changes.

Little is known about the life cycle of *P. homopolare* and its pathology in different hosts. It has been suggested that *P. homopolare* progresses to a chronic infection in hosts with only a short window of time during which the gametocytemia is high enough to infect mosquitoes making it difficult to detect^29^. Disease dynamics of avian haemosporidians include a brief pre-patent period in which the parasites could be found only in the host tissues, followed by a patent stage that starts with a relatively short period of acute infection with high parasitemia. Then, after the acute infection, an indefinite period of chronic infection with low parasitemia is followed^35–37^. Add to this pattern the possibility that, in the Holarctic region, patent *Plasmodium* spp. infections could be affected by the seasons where some parasite species are unlikely to be detected in the hosts in early spring and/or late fall using microscopy^36^. In addition, likely there is a low rate of parasite transmission during the winter (the only time when the California Condors can be sampled because they congregate to reproduce) given the low population densities of vectors^38^. All these factors make it difficult to observe patent infections and to assess the Condor exposure to *P. homopolare* as part of a surveillance program*.*

Nevertheless, there are two lines of evidence suggesting that the *P. homopolare* infections reported here were subpatent: (1) there was no evident sign of pathogenesis associated with these infections in Condors, and (2) nested PCR was required to detect them. Subpatent infections (usually submicroscopic) make it difficult to determine the prevalence of haemosporidian parasites. In such cases, parasites can only be detected by PCR using genes with high copy numbers like the one used in this study (*cytb*^39^). However, the finding of three infected individuals with the same parasite lineage during the winter season suggests that its transmission is active.

Overall, the low parasite prevalence found in Condors could be explained, at least in part, by (a) the short window of time during which the parasitemia is high enough even to be detectable by PCR, (b) the timing when the samples were collected given that all birds were captured during the winter season (from November to March, Supporting information Table S1), and (c) the California condor could be an incidental host of *P. homopolare* that is exposed multiple times to the same parasite because it is circulating in other species in the area^30^. These scenarios can only be explored if there is sampling during other times of the year. However, as previously stated, sampling year around is particularly difficult given the Condor behavior and broad geographic distribution. Nevertheless, this study shows that the California Condors are at risk of vector-borne pathogens that can spillover from other species locally.

In conclusion, considering the loss of genetic diversity in California Condor populations and environmental stressors such as lead poisoning, this endangered species could be vulnerable to infectious diseases, in particular, haemosporidian parasites. Fortunately, the parasite species detected at this time has no observable consequences to the Condor populations. Thus, active pathogen surveillance should be an essential part of conservation efforts to mitigate early the impact of infectious diseases, an increasingly recognized cause of global wildlife extinctions worldwide^40^, particularly in avian populations considered vulnerable or endangered.

## Material and methods

### Study area and samples

Since 1996, The Peregrine Fund has released captive-bred condors in northern Arizona; each condor has been identified by a studbook number assigned at fledging^41^. The study area is located atop Vermillion Cliffs and in view of the Kaibab Plateau to the west, approximately 80 km north of the south rim of the Grand Canyon (36° N. Lat, 112° W. Long). Condors were captured in a “walk-in” chain-link trap measuring approximately 6.1 m by 12.19 m. Pre-baiting with bovine calf carcasses encouraged condors to enter and exit the trap freely. Calf carcasses were donated from Phoenix Dairies following state and federal standards. Condors were observed from a blind, and the door to the trap was closed by means of a hand-operated cable and pulley system. Then, each target individual was captured inside the trap with a hand net and transported to a nearby processing area. From one to three people held the condor while a fourth drew 1–3 ml of blood from the medial-tarsal vein using a 22-ga needle and heparinized tubes for sample storage. Fifty μg of whole blood from each condor was transferred to a vial containing 250 μl of 0.35 molar HCl for lead analysis in the field. Then, 50 to 100 μl of whole blood was preserved in protein saver cards (Whatman 903, Whatman, Cardiff, UK) for molecular analysis. In total, 208 condors from 2008 to 2018 were screened for haemosporidian parasites (20 condors in 2008, 55 in 2014/15, 66 in 2016/17, and 67 in 2018, Supporting information Table S1).

### Ethical statement and permits

As part of the lead surveillance program, bird capture and manipulation were carried out in a way that reduced stress following standard and published protocols^4–8^. All methods were performed in accordance with the relevant guidelines and regulation. Protocols and sample collection were approved by US Fish and Wildlife Service under permit USFWS TE25609A-2.

### Molecular diagnostic of haemosporidian parasites

DNA from whole blood was extracted using QIAamp DNA Micro Kit (QIAGEN GmbH, Hilden, Germany). Then, each sample was screened for haemosporidian parasites by using a nested polymerase chain reaction (PCR) protocol that targets the parasite mitochondrial cytochrome b gene (*cytb*, 1,131 bp) using the primers described by Pacheco et al.^39,42^. Primary PCR amplifications were carried out using a 50 µl volume reaction using 5-8 µl of total genomic DNA, 2.5 mM MgCl2, 1 × PCR buffer, 0.25 mM of each deoxynucleoside triphosphate, 0.4 µM of each primer, and 0.03 U/µl AmpliTaq polymerase (Applied Biosystems, Thermo Fisher Scientific, USA). *Cytb* external primers were forward AE298 5′-TGT AAT GCC TAG ACG TAT TCC 3′ and reverse AE299 5′-GT CAA WCA AAC ATG AAT ATA GAC 3′. The primary PCR conditions were: A partial denaturation at 94 °C for 4 min and 36 cycles with 1 min at 94 °C, 1 min at 53 °C and 2 min extension step at 72 °C. We added a final extension step of 10 min at 72 °C in the last cycle. Nested PCRs were also carried out in 50 µl volume reaction using 1 µl of the primary PCRs, 2.5 mM MgCl2, 1 × PCR buffer, 0.25 mM of each deoxynucleoside triphosphate, 0.4 µM of each primer, and 0.03 U/µl AmpliTaq polymerase. *Cytb* internal primers were forward AE064 5′-T CTA TTA ATT TAG YWA AAG CAC 3′ and reverse AE066 5′-G CTT GGG AGC TGT AAT CAT AAT 3′. The nested PCR conditions were the same used for the primary PCR but with an annealing temperature of 56 °C. After electrophoresis, all PCR amplified products (50 µl) were excised from the gels, purified by the QIAquick Gel Extraction Kit (QIAGEN GmbH, Hilden, Germany) and both strands for the *cytb* fragments were directly sequenced using an Applied Biosystems 3730 capillary sequencer. By careful inspections of each electropherogram, each sequence was checked for mixed infections. *Cytb* sequences obtained in this study were identified as *Plasmodium* using BLAST^17^, and deposited in GenBank under the accession numbers MT341242-MT341244.

### Phylogenetic and population analyses

In this study, three nucleotide alignments were performed using ClustalX v2.0.12 and Muscle as implemented in SeaView v4.3.5^43^. A first alignment (1,012 bp out of the 1,134 bp of *cytb* gene, excluding gaps) was constructed with 35 sequences. This alignment included the sequences obtained in this study (N = 3) as well as complete *cytb* sequences (N = 32) from well-known parasite species based on morphology^36,44^ that were available in GenBank^45^ at the time of this study. A second alignment (N = 45) was done using the small commonly used *cytb* fragment (464 bp) from well-known parasite species based on morphology^28,33^. The use of a larger *cytb* fragment is expected to yield a better phylogenetic signal than the one usually amplified in these types of studies^28,33^, but includes less information in terms of isolates (N = 35 vs. 45). In both alignments, the included species belonged to three genera (*Leucocytozoon*, *Haemoproteus*, and *Plasmodium*). Finally, a third alignment (411 bp excluding gaps) was done, including all 76 partial *cytb* sequences of *P. homopolare* available in GenBank^45^ and MalAvi^28^ databases. In this alignment, one sequence of *Plasmodium parahexamerium* was also included for being one of the closest taxa to *P. homopolare* given the data available.

Three phylogenetic hypotheses were inferred based on those alignments. The first (1,012 bp) and second (464 bp) alignments were used to infer the relationships between the *Plasmodium* parasites, including the lineages found in the condors. Trees were estimated using the Bayesian method implemented in MrBayes v3.2.6 with the default priors^46^ and the best model that fit the data (general time-reversible model with gamma-distributed substitution rates and a proportion of invariant sites, GTR + Γ + I). This model was the one with the lowest Bayesian Information Criterion (BIC) scores, as estimated by MEGA v7.0.14^47^. Bayesian support was inferred for the nodes in MrBayes by sampling every 1,000 generations from two independent chains lasting 2 × 10^6^ Markov Chain Monte Carlo (MCMC) steps. The chains were assumed to have converged once the value of the potential scale reduction factor (PSRF) was between 1.00 and 1.02, and the average SD of the posterior probability was < 0.01^46^. Once convergence was reached as a “burn-in,” 25% of the samples were discarded. *Leucocytozoon* species were used as the outgroup in these phylogenies. Genbank accession numbers and the MalAvi lineage code of all sequences used in the analyses are shown in the phylogenetic trees. The second alignment (464 bp) was also used to estimate the pairwise evolutionary divergences between *Plasmodium* species (N = 12) closely related to *P. homopolare* by using the p-distance method as implanted in MEGA v7.0.14^47^.

In order to compare all *P. homopolare* haplotypes available, including those found in this study, a phylogenetic tree was estimated with the third alignment (411 bp) using the Maximum Likelihood method with a General Time Reversible model. The robustness was assessed by bootstrap on 1,000 replicates. All calculations were performed using MEGA v7.0.14^47^. The tree was drawn to scale, with branch lengths measured in the number of substitutions per site. Host species names and Genbank accession numbers of the *P. homopolare* sequences used in the analysis are shown in the phylogenetic tree. In this case, *P. parahexamerium* was used as the outgroup.

## Supplementary information

Supplementary Tables.

Supplementary Information.
