# Supplementary material for: The endangered California Condor (Gymnogyps californianus) population is exposed to local haemosporidian parasites
Source: Sci Rep. 2020 Oct 21;10:17947. doi: 10.1038/s41598-020-74894-0 (PMC7578645; doi:10.1038/s41598-020-74894-0)
Supplement: Supplementary file 2 — Supplementary Information. [file 41598_2020_74894_MOESM2_ESM.docx]

**Supplementary material for**

**The endangered California Condor (*Gymnogyps californianus*) population is exposed to local haemosporidian parasites**

M. Andreína Pacheco^1,*^, Chris N. Parish^2^, Timothy J. Hauck^2,^ Roberto F. Aguilar^3^, Ananias A. Escalante^1,*^

^1^Biology Department/Institute of Genomics and Evolutionary Medicine (iGEM), Temple University, Philadelphia, PA 19122-1801, USA.

^2^The Peregrine Fund, 5668 West Flying Hawk Lane, Boise, Idaho 83709, USA

^3^Tucson Wildlife Center, 13275 East Speedway, Tucson, Arizona 85748-7125, USA

**^*^Corresponding author:**

M. Andreína Pacheco, [Maria.Pacheco@temple.edu](mailto:Maria.Pacheco@temple.edu)

Ananias A. Escalante, [Ananias.Escalante@temple.edu](mailto:Ananias.Escalante@temple.edu)

**Supplementary Table S1 (Excel file). California Condor (*Gymnogyps californianus*) samples collected in Arizona from 2008 to 2018.** In red are indicated the positive samples obtained in this study.

**Supplementary Table S2 (Excel file). List of *P. homopolare* haplotype sequences included in Figure 2.** Host names, Genbank accession numbers, and origin of the sequences are provided.

**Supplementary Table S3 (Excel file). Total of infected individuals with haplotype H1 and percentage per avian order and family.**
